# Molecular signatures of inherited and acquired sporadic late onset nemaline myopathies

**DOI:** 10.1186/s40478-023-01518-9

**Published:** 2023-01-26

**Authors:** Stefan Nicolau, Aneesha Dasgupta, Surendra Dasari, M. Cristine Charlesworth, Kenneth L. Johnson, Akhilesh Pandey, Jason D. Doles, Margherita Milone

**Affiliations:** 1 Department of Neurology, Mayo Clinic, 200 First St. SW, Rochester, MN 55905 USA; 2 Center for Gene Therapy, Nationwide Children’s Hospital, Columbus, OH 43205 USA; 3 Department of Biochemistry and Molecular Biology, Mayo Clinic, Rochester, MN 55905 USA; 4 Department of Anatomy, Cell Biology and Physiology, Indiana University School of Medicine, Indianapolis, IN 46202 USA; 5 Indiana Center for Musculoskeletal Health, Indiana University School of Medicine, Indianapolis, IN 46202 USA; 6 Department of Quantitative Health Sciences, Mayo Clinic, Rochester, MN 55905 USA; 7 Proteomics Core, Medical Genomics Facility, Mayo Clinic, Rochester, MN 55905 USA; 8 Department of Laboratory Medicine and Pathology, Mayo Clinic, Rochester, MN 55905 USA; 9 Manipal Academy of Higher Education (MAHE), Manipal, Karnataka 576104 India

**Keywords:** Nemaline myopathy, NEB, ACTA1, Histology, Proteomics, Transcriptomics, RNA-seq

## Abstract

Acquired sporadic late onset nemaline myopathy (SLONM) and inherited nemaline myopathy (iNM) both feature accumulation of nemaline rods in muscle fibers. Unlike iNM, SLONM is amenable to therapy. The distinction between these disorders is therefore crucial when the diagnosis remains ambiguous after initial investigations. We sought to identify biomarkers facilitating this distinction and to investigate the pathophysiology of nemaline rod formation in these different disorders. Twenty-two muscle samples from patients affected by SLONM or iNM underwent quantitative histological analysis, laser capture microdissection for proteomic analysis of nemaline rod areas and rod-free areas, and transcriptomic analysis. In all iNM samples, nemaline rods were found in subsarcolemmal or central aggregates, whereas they were diffusely distributed within muscle fibers in most SLONM samples. In SLONM, muscle fibers harboring nemaline rods were smaller than those without rods. Necrotic fibers, increased endomysial connective tissue, and atrophic fibers filled with nemaline rods were more common in SLONM. Proteomic analysis detected differentially expressed proteins between nemaline rod areas and rod-free areas, as well as between SLONM and iNM. These differentially expressed proteins implicated immune, structural, metabolic, and cellular processes in disease pathophysiology. Notably, immunoglobulin overexpression with accumulation in nemaline rod areas was detected in SLONM. Transcriptomic analysis corroborated proteomic findings and further revealed substantial gene expression differences between SLONM and iNM. Overall, we identified unique pathological and molecular signatures associated with SLONM and iNM, suggesting distinct underlying pathophysiological mechanisms. These findings represent a step towards enhanced diagnostic tools and towards development of treatments for SLONM.

**Supplementary Information:**

The online version contains supplementary material available at 10.1186/s40478-023-01518-9.

## Introduction

Inherited nemaline myopathies (iNM) are a genetically heterogeneous group of myopathies so far known to be caused by mutations in 13 different genes (Table 1) [1–3]. In some patients with classic iNM, however, a specific molecular defect has yet to be identified, and it is therefore likely that more causative genes and non-coding mutations remain undiscovered. In addition, nemaline rods can occur, although not as the prevalent morphological abnormality, in the setting of protein defects classically associated with myofibrillar pathology or other morphological features (e.g. rimmed vacuoles) [4–7].

**Table 1:** Classification of nemaline myopathies

	Gene	Protein
iNM	TPM3	α-tropomyosin
	NEB	Nebulin
	ACTA1	α-actinin
	TPM2	β-tropomyosin
	TNNT1	Troponin T1
	KBTBD13	Kelch-repeat and BTB domain containing 13
	CFL2	Cofilin-2
	KLHL40	Kelch-like family member 40
	KLHL41	Kelch-like family member 41
	LMOD3	Leiomodin-3
	MYPN	Myopalladin
	RYR1	Ryanodine receptor 1
	ADSSL1	Adenylosuccinate synthase-like 1

	Subtype	
SLONM	With monoclonal gammopathy	
	With HIV infection	
	Without monoclonal gammopathy or HIV infection	

Sporadic late onset nemaline myopathy (SLONM), on the other hand, is an acquired myopathy, sometimes associated with monoclonal gammopathy of unknown significance (MGUS), or less frequently with biclonal gammopathy, or HIV infection (Table 1) [8–10]. While the pathophysiology of SLONM is poorly understood, recent studies suggest that SLONM is a potentially treatable disease [8, 9, 11].

Muscle biopsies of both iNMs and SLONM feature nemaline rods as the main pathological finding, which represent aggregates of Z-disc and thin filament-related proteins [12]. These proteins include α-actinin, actin, tropomyosin, myotilin, γ-filamin, cofilin-2, telethonin and nebulin. Nemaline rods appear red on the Gomori trichrome stain and have an electron-dense lattice-like structure on electron microscopy. Continuity can be observed between rods and Z-discs. Nemaline rods can be cytoplasmic and localize within the I band or extend for many sarcomeres. Nemaline rods can also be intranuclear in some types of nemaline myopathy (e.g. *ACTA1*-related iNM) [2]. The role of nemaline rods in iNM pathophysiology has been debated, as mutations in the same genes can cause myopathy without nemaline rods. Recently, impairment of myosin stabilization conformation state and increase energy consumption of resting muscle fibers has been demonstrated in iNM [13].

In some patients, the accurate distinction between iNM and SLONM poses challenges, yet this distinction is critical as SLONM can respond to immunomodulatory therapy or bone marrow transplantation, while iNM does not. Despite iNMs being classified as congenital myopathies and frequently manifesting in infancy or childhood, expanded genetic testing has led to a growing number of reports of adult presentations [14, 15]. On the other hand, SLONM, which presents in adulthood and often subacutely, can have a chronic course with a limb-girdle phenotype mimicking an inherited myopathy [16], or may lack associated MGUS or HIV infection. The clinical and pathological features of iNM and SLONM can thus overlap. In this study, we leveraged pathological, genetic, and proteomic studies to identify biomarkers that could help distinguish SLONM from iNM and to investigate pathophysiological mechanisms leading to nemaline rod formation in these two distinct muscle disorders. We employed targeted proteomic analyses to analyze the composition of nemaline rods, as well as bulk transcriptomics to assess broader alterations in gene expression across the two disorders.

## Materials and methods

### Patients

Patients with a clinical-pathological or clinical-pathological-genetic diagnosis of SLONM or iNM, respectively, who had undergone muscle biopsy as part of their diagnostic investigations were identified through a search of medical records. Muscle samples were used for histological, proteomic and transcriptomic analysis.

### Genetic testing

Sequencing of 123 genes (Additional file 2: Table S1) known to cause inherited myopathies was performed in all patients (Invitae corporation, San Francisco, CA). This gene panel included all genes so far known to cause iNM or nemaline rods in association with other structural abnormalities.

### Histology

**Fig. 1:**
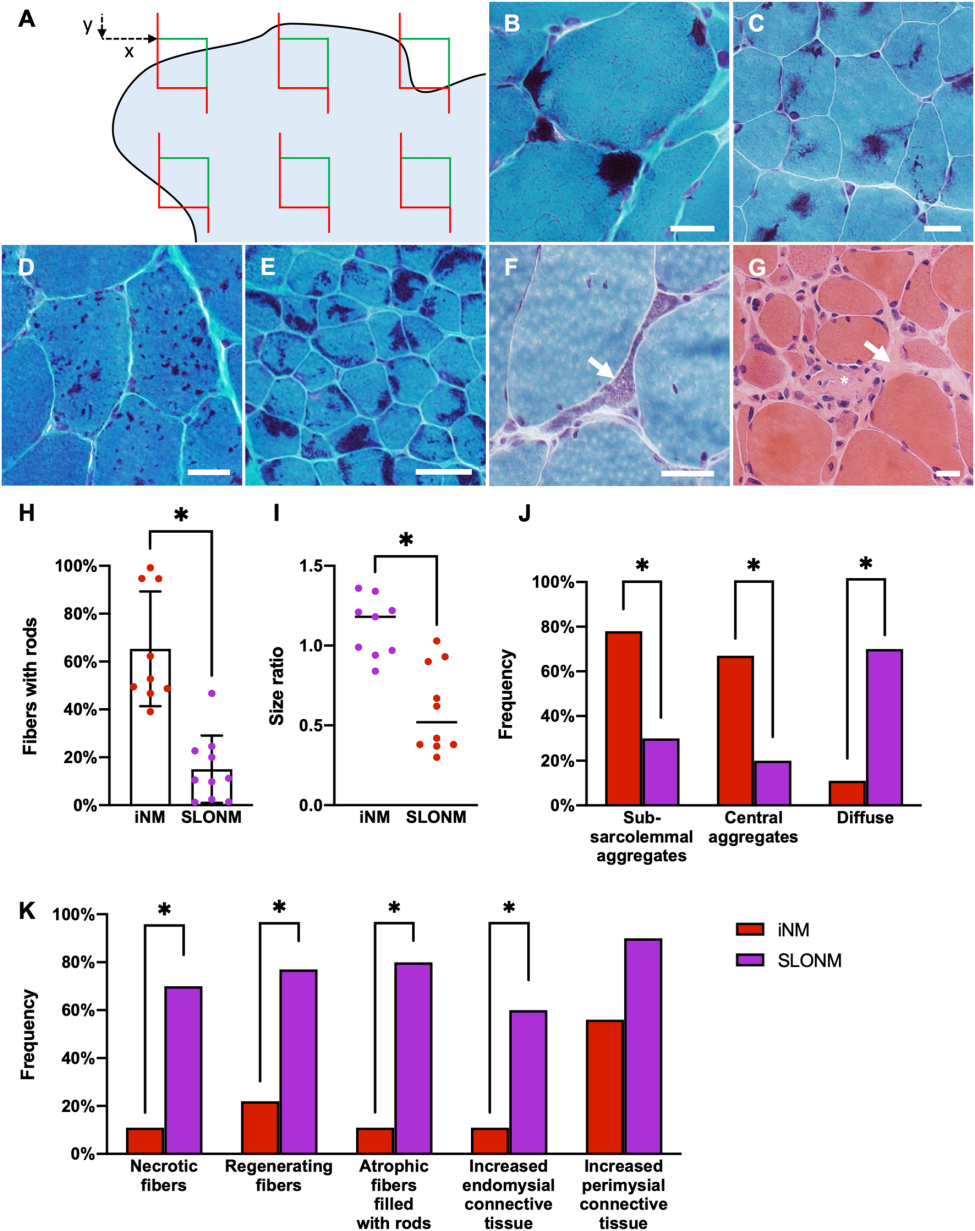
Quantitative myopathological analysis of inherited and sporadic late-onset nemaline myopathies (**A**) Unbiased quantitative myopathological analysis was performed by systematic random sampling of digitized images of muscle sections. The distribution of nemaline rods in muscle fibers was categorized as (**B**) subsarcolemmal aggregates, (**C**) central aggregates, or (**D**) diffuse. (**E**) In some inherited nemaline myopathy samples, > 90% of fibers contained rods. (**F**) Atrophic fibers filled with rods, (**G**) necrotic fibers (*), and increased endomysial connective tissue (arrow) were seen almost exclusively in sporadic late-onset nemaline myopathy. (**H**) In inherited nemaline myopathy (iNM), fibers containing nemaline rods were more frequent than in sporadic late onset nemaline myopathy (SLONM). Bars represent mean ± standard deviation. (**I**) The diameters of fibers containing rods were  larger in iNM than in SLONM. Lines represent the mean of each group. (**J**) In iNM, rods were most often found in aggregates, whereas they were diffusely distributed in SLONM. (**K**) Necrotic fibers, regenerating fibers, atrophic fibers filled with rods, and increased endomysial connective tissue were all more common in SLONM. A–F: modified Gomori trichrome stain, G: hematoxylin and eosin, scale bar = 20 µm in all panels, *p < 0.05

Muscle biopsy specimens were processed for routine diagnostic histological analysis as previously described [17]. Quantitative analyses were performed on 10 µm frozen sections stained with modified Gomori trichrome. Slides were scanned at 40 × magnification using a Zeiss Axio Scan.Z1 (Carl Zeiss AG, Oberkochen, Germany). Unbiased systematic random sampling was achieved by overlaying a counting frame at regular intervals on the scanned images, with randomly chosen initial x and y offsets (Fig. 1A). Given the significant variability in muscle fiber diameter and biopsy size between samples, the counting frame size (100 × 100 µm, 200 × 200 µm, or 400 × 400 µm) and spacing (800 to 2000 µm) were selected on a sample-by-sample basis to achieve approximately 25 counting sites per sample and 4–20 fibers per site.

The number of muscle fibers with and without nemaline rods and their minimum cross-sectional diameters were measured manually (ImageJ 1.52p, National Institutes of Health). The distribution of nemaline rods in each sample was classified as central aggregates, subsarcolemmal aggregates, or diffuse (Fig. 1B–F). When a single biopsy displayed multiple distributions of nemaline rods, such findings were recorded. Muscle fiber necrosis, regeneration, and presence of increased connective tissue were assessed on review of entire sections stained with hematoxylin and eosin and modified Gomori trichrome (Fig. 1G).

### Laser capture microdissection

10 µm frozen sections of tissue collected on polyethylene naphthalate slides were stained with modified Gomori trichrome. The slides underwent UV laser capture microdissection by the laser pressure catapulting method on the Zeiss PalmMicrobeam LPC system using the PALM Robo software (Zeiss, White Plains, NY). Nemaline rods could not be individually isolated due to their small size, and microdissection therefore focused on capturing areas of muscle fibers containing high concentrations of rods. Equal areas of rod-free fibers were also collected. Nuclei in sample areas were avoided if possible.

Areas of interest were laser catapulted into the caps of 0.5 ml tubes, containing 35 µL of digest buffer consisting of 100 mM Tris, pH 8.2 and 0.005% Zwittergent 3–16 (Sigma, St. Louis, MO). Immediately upon collection, the dissected tissue samples were collected in the bottom of the tubes by centrifugation at 14000 g for 2 min, then frozen at −80 °C.

When all tissue was collected, samples were thawed and ProteaseMAX (Promega, Madison, WI), a surfactant that enhances trypsin digest, was added to a final concentration of 0.05%. Sample tubes were shaken at 400 rpm at ambient temperature for 30 min to extract and solubilize protein. After reduction and alkylation with 10 mM dithiothreitol and iodoacetamide, respectively, proteins were digested with trypsin (Promega) at 37 °C for 18 h. Samples were acidified with trifluoroacetic acid (Thermo Fisher Scientific, Waltham, MA) and transferred to sample vials for mass spectrometry analysis.

### Mass spectrometry

Nano-scale liquid chromatography tandem mass spectrometry (nLC-MS/MS) data was acquired on an Orbitrap Q-Exactive mass spectrometer (Thermo Fisher, Bremen, Germany), interfaced with a Dionex 3000 RSLC liquid chromatography system. Peptides were separated on a 33 cm long PicoFrit fused silica column (75 µm internal diameter) self-packed with Acclaim RSLC 2.2 µm, 120 Å C_18_ stationary phase using a gradient of 2%B mobile phase to 40%B at 100 min, flow rate of 250nL/min, followed by a 5 min ramp to 85%B held for 6 min, then re-equilibrated at 2%B. Mobile phase A was 2% acetonitrile in water with 0.2% formic acid. Mobile phase B was 80% acetonitrile, 10% isopropanol, 10% water with overall 0.2% formic acid. Samples were loaded via autosampler and pre-concentrated from a 20µL loop on a 0.33µL EXP2 stem trap packed with Halo 2.7 µm Pep ES-C18 (Optimize Technologies, Oregon City, OR) for 6 min at 8µL/min before switching the trap in-line with the separation column during the gradient.

Mass spectrometry data was collected in a data dependent acquisition mode. MS1 data was collected from m/z 340–1600 Thompson (Th) using resolving power of 70,000 (fwhm at m/z 200), automatic gain control (AGC) of 3E6, with a maximum ionization time (maxIT) of 150 ms. MS2 spectra were collected at 17,500 resolving power from the top 20 MS1 precursors with charge states (z) of 2–5, using a precursor isolation window of 2.0 Th, with maxIT = 70 ms, normalized collision energy of 27, minimum precursor intensity of 1.2E5, AGC = 8,500, and MS2 first mass of 140Th. Precursor masses selected for MS2 spectra were subsequently excluded for 20 s.

### Immunofluorescence studies

Frozen skeletal muscle sections  (8 µm) were post-fixed in 4% paraformaldehyde for 5 min at room temperature prior to immunostaining with laminin (L0663, Sigma, dilution 1:250) and human kappa light chain (NBP2-69235, Novus Biologicals, Centennial, CO, dilution 1:200) antibodies as previously described [18].

### Immunohistochemical studies

Frozen skeletal muscle sections (10 µm) were post-fixed in acetone at −20 °C for 10 min prior to immunostaining with major histocompatibility complex class I (MHC-I) antibodies (W6/32, Dako, Glostrup, Denmark, dilution 1:2000) as previously described [19].

### RNA isolation and RNA sequencing

Total RNA was extracted from muscle tissues using the TRIzol reagent (Thermo Fisher Scientific, Carlsbad, CA) as previously described [20] and was isolated using RNeasy columns (Qiagen, Germantown, MD ). Three clinical muscle biopsies demonstrating no histological abnormalities were used as controls. RNA integrity was verified using a Fragment Analyzer (Agilent, Santa Clara, CA). Library preparation, sequencing and analysis were performed at the Mayo Clinic Medical Genome Facility as previously described [21]. Briefly, mRNA libraries were generated using the TruSeq Stranded mRNA kit (Illumina, San Diego, CA), then pooled and sequenced on Illumina High Seq 4000 using 100 bp paired-end reads and 8 samples per lane.

### Statistical analysis of histology data

Data are expressed as means and standard deviations. Comparisons were performed using the Student T and Chi-square tests. A *p* value of < 0.05 was considered significant for all analyses.

### Bioinformatics of label-free proteomics

We followed a modified version of a previously published [22] protocol for processing the raw nLC-MS/MS data to generate protein identifications and perform differential expression analysis. The raw data files were searched against the SwissProt human protein sequence database (downloaded on 1/17/2022) using MaxQuant (version 2.0.3.0). The software was instructed to append reversed protein entries to the database for estimating protein and peptide false discovery rates (FDRs). MaxQuant was configured to use the default Orbitrap parameters augmented to search for the following variable posttranslational modifications: carbamidomethylation of cysteine, oxidation of methionine, formation of n-terminal pyroglutamic acid, and deamidation of asparagine. Peptides were identified at an FDR of < 0.01. Resulting protein identifications with at least one unique peptide identification were quantified using the corresponding peptide MS1 intensities. The resulting matrix of raw protein intensities in each sample was processed to impute missing values using the lower tail method by first determining the overall distribution of protein intensities in all samples, then imputing missing values by randomly selecting an intensity from the bottom five percentiles of the overall protein intensity distribution. Processed protein intensities were log_2_ transformed and normalized using the quantile method, and these values were utilized to compare the relative abundance of proteins between any two groups. For this, each protein’s intensities observed across any two groups were modeled using a Gaussian-linked generalized linear model. A null model was also created for each protein by removing the sample group information. ANOVA contrasts were utilized to assess the significance of relationship between each protein’s intensities versus the two groups over the null model. Resulting differential expression p-values were adjusted using the Benjamini–Hochberg method. Proteins with an adjusted differential expression *p* value of < 0.05 and an absolute log_2_ (fold change) of > 1 were considered as significantly different between the two groups.

### Protein set enrichment analysis

Differential expression results from any two-group comparison were subjected to the gene set enrichment analysis as previously described. In brief, each protein was mapped to its corresponding gene and a rank score was computed using the −log_2_ (differential expression *p* value) and sign of the log_2_ (fold change). The gene symbols and the rank scores corresponding to each protein were loaded into the Broad Institute Gene Set Enrichment Analysis software and analyzed using the Preranked method [23] configured to use Hallmark, Reactome, KEGG and Biocarta pathways. Gene sets with an FDR-adjusted *q* value ≤ 0.05 were selected for further interpretation.

### Immunoglobulin light chain proteomic analysis

We utilized a previously published method [24, 25] to identify the constant and variable regions of immunoglobulin heavy chains (HC) and light chains (LC) from nLC-MS/MS data. In brief, MyriMatch [26] software matched the raw nLC-MS/MS spectra against a custom-built human protein sequence database containing the SwissProt human reference proteome augmented with HC and LC variable region sequence templates obtained from AL-Base [27] and IMGT [28]. Reversed protein sequence entries were appended to the protein database to estimate peptide and protein FDRs. MyriMatch was configured to use the same variable posttranslational modifications and mass tolerances described above. IDPicker software [29] filtered the resulting peptide identifications at an FDR of < 0.01. Protein identifications with at least two unique peptide identifications were considered to be present in the sample. Total number of spectra matching to the HC and LC constant and variable regions in each sample were extracted and compared against any two experimental groups using a quasi-likelihood method described previously [30]. HC and LC constant and variable regions that had an adjusted differential expression *p* value of < 0.05 between any two groups were considered differentially expressed.

### Bioinformatics of transcriptomic data

Reads were aligned to the hg38 reference build of the human genome using MAP-Rseq v.3.1.4. Tibco Spotfire software and Ingenuity Pathway Analysis were utilized to analyze the resulting data. Genes with an adjusted differential expression *p* value of < 0.05 and an absolute log_2_(fold change) of > 1 were considered as differentially expressed between iNM and SLONM.

## Results

### Patients

Thirteen patients with SLONM and 9 patients with iNM were included in this study. Demographics and key clinical and genetic characteristics are summarized in Table 2. Their median ages were 64 and 11 years, respectively. Four iNM patients were older than 18 years. Causative genes were identified in all iNM patients: *ACTA1* (n = 5), *NEB* (n = 3) and *TNNT1* (n = 1). All iNM patients diagnosed in adulthood were tested for monoclonal gammopathy and found to be negative. Nine of 13 SLONM patients had an underlying monoclonal gammopathy. All SLONM patients were HIV-negative and none had a reportable variant in genes known to cause iNM.

**Table 2:** Clinical features of inherited and sporadic late-onset nemaline myopathy patients

Pt	Age at onset	Age at diagnosis	Distribution of weakness	Severity	
Inherited nemaline myopathy					Gene
1	1st decade	31	Scapulo-peroneal	Mild	*ACTA1*
2	1st decade	58	UL, LL, prox > dist	Moderate	*ACTA1*
3	Early 70 s	73	Generalized	Moderate-severe	*NEB*
4	1st decade	17	UL, prox and axial	Mild	*ACTA1*
5	Prenatal	3	Generalized	Severe	*ACTA1*
6	Unknown	9	Unknown	Unknown	*NEB*
7	5	7	LL prox > dist	Unknown	*NEB*
8	Unknown	12	Unknown	Unknown	*ACTA1*
9	Unknown	2	Unknown	Unknown	*TNNT1*
Sporadic late-onset nemaline myopathy					Monoclonal gammopathy
10	56	57	UL, LL, prox > dist, axial	Moderate	Absent
11	58	61	UL, prox; LL, prox > dist, axial	Moderate	IgG kappa
12	65	67	LL, prox	Moderate	Free lambda LC, ↑
13	45	46	LL > UL, prox	Moderate-severe	IgG kappa
14	59	63	UL, LL, axial	Moderate	IgG kappa
15	63	64	Axial > UL and LL, prox	Moderate	Absent
16	70	77	UL > LL, prox > dist	Mild-moderate	IgG kappa + IgG lambda
17	65	67	UL, axial	Mild-moderate	IgG lambda
18	60	73	UL > LL, axial	Moderate-severe	Absent
19	63	67	LL, prox > dist; LL > UL, prox	Mild-severe	IgG kappa
20	46	56	UL, LL, axial	Severe	IgG lambda
21	56	59	UL, LL, axial	Moderate-severe	IgG kappa + IgG lambda

*Dist* distal,*LC* light chain, *LL* lower limbs, *prox* proximal; *UL* upper limbs

Severity was based on strength of weak muscles according to Medical Research Council (MRC): mild (MRC 4); moderate (MRC 3–3.5); severe (MRC 0 to < 3)

### Histological analysis identifies morphological differences between SLONM and iNM

Histological analysis was performed on all 22 diagnostic muscle biopsy samples (13 SLONM and 9 iNM). Findings are summarized in Table 3. The mean proportion of fibers harboring nemaline rods was significantly higher in iNM than in SLONM (65 and 11%, respectively, *p* < 0.0001). In three iNM samples (33%), all of them pediatric, > 90% of fibers contained rods (Fig. 1E). In SLONM, fibers with rods were smaller than those without (mean ratio 0.60), while in iNM fibers with rods were larger (mean ratio 1.12). In 10 SLONM patients (77%), rods were spread diffusely within the individual fibers, while rods occurred in central or subsarcolemmal aggregates in all iNM samples. Most iNM samples displayed both central and subsarcolemmal aggregates. There were no significant differences in any of these parameters between patients with *ACTA1* and *NEB* mutations, but comparisons were limited due to the small sample size. Atrophic fibers filled with rods were seen in 8 SLONM samples (80%), where they represented 8–65% of all fibers with rods. By contrast, they were only seen in a single iNM sample (11%), in which they accounted for 1% of fibers with rods. Necrotic fibers and increased endomysial connective tissue were both more frequent in SLONM than iNM. The single iNM patient with necrotic fibers was also the oldest patient, aged 73. Among SLONM patients, there was a trend towards an increased proportion of fibers containing nemaline rods in those without monoclonal gammopathy (mean 23% vs. 10%), but this did not reach statistical significance. A minimal perivascular inflammatory reaction was observed in a single SLONM sample. No amyloid deposition was observed.

**Table 3:** Histological features of inherited and sporadic late-onset nemaline myopathies

		iNM	SLONM
Proportion of fibers with nemaline rods, mean (range)		53% (39–99%)	11% (1–47%)
Size ratio of fibers with and without rods, mean (range)		1.12 (0.84–1.36)	0.60 (0.30–1.03)
Atrophic fibers filled with rods, % of samples		11%	77%
Necrotic fibers, % of samples		11%	62%
Regenerating fibers, % of samples		22%	77%
Increased endomysial connective tissue, % of samples		11%	62%
Increased perimysial connective tissue, % of samples		56%	93%
Inflammation, % of samples		0%	8%
Distribution of nemaline rods within muscle fibers, % of samples*	Diffuse	11%	77%
	Central aggregates	67%	23%
	Subsarcolemmal aggregates	78%	23%

*More than one distribution may be seen in each sample, *iNM* inherited nemaline myopathy, *SLONM* sporadic late-onset nemaline myopathy

### Proteomic analysis reveals differential protein abundance between SLONM and iNM

**Fig. 2:**
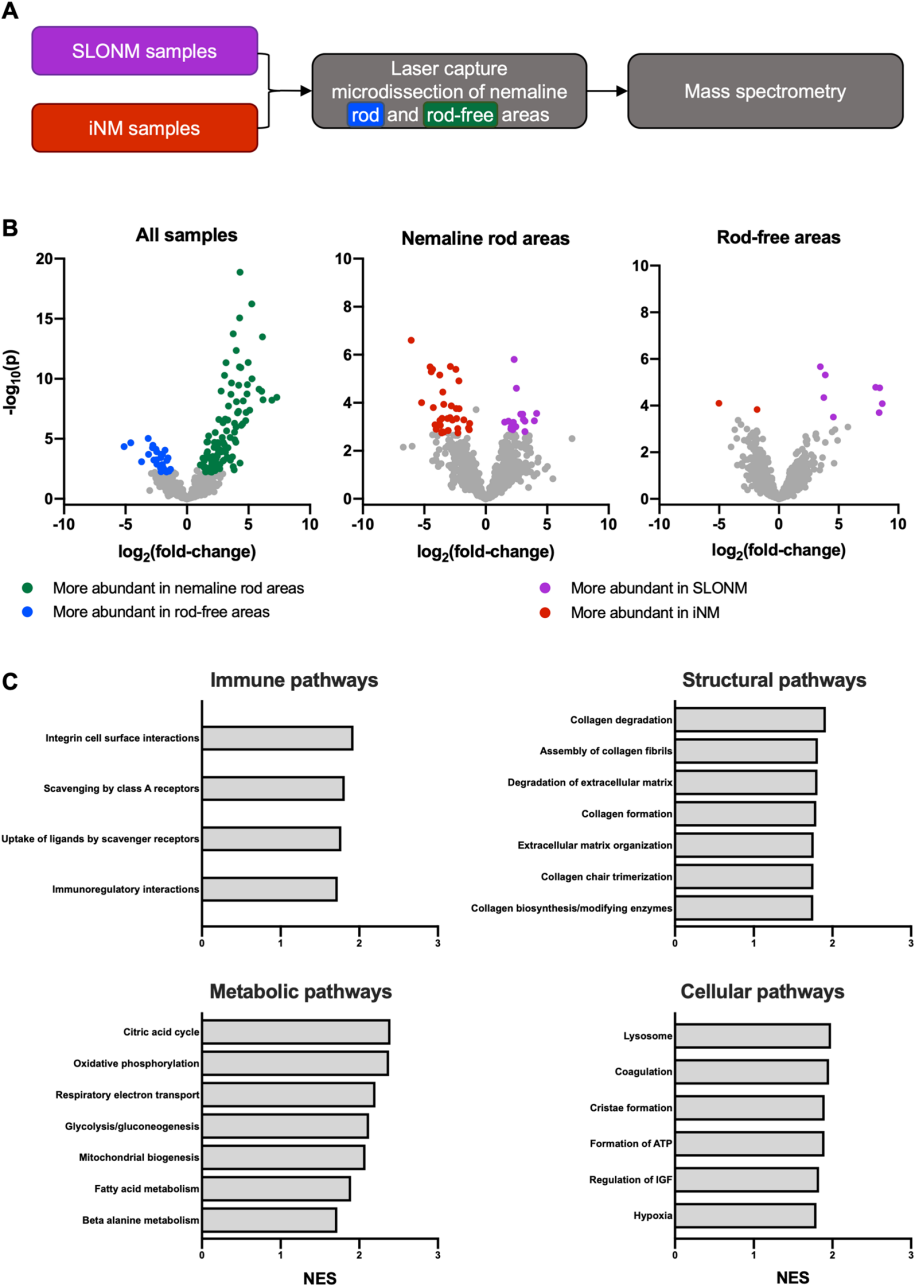
Proteomic analysis of inherited and sporadic late-onset nemaline myopathies after laser capture microdissection (**A**) Nemaline rod and rod-free areas from sporadic late onset nemaline myopathy (SLONM) and inherited nemaline myopathy (iNM) samples were subjected to laser capture microdissection and subsequently processed for proteomic analysis. (**B**) Volcano plots demonstrate differentially expressed proteins between rod and rod-free areas, as well as between SLONM and iNM samples. The left panel depicts protein abundance in nemaline rod areas relative to rod-free areas across all samples, whereas the center and right panels depict protein abundance in SLONM relative to iNM in rod and rod-free areas, respectively. (**C**) Comparison of the rod areas of SLONM and iNM showed enrichment of targets corresponding to several immune, structural, metabolic, and cellular pathways

Proteomic analysis by laser capture microdissection was performed on 20 biopsies (11 SLONM and 9 iNM, Fig. 2A). Two biopsies could not be included due to lack of tissue availability. Areas of muscle fibers containing clusters of nemaline rods were targeted for laser capture microdissection, with an average of 225 rod regions cut per sample (corresponding to an area of ~ 0.09 mm^2^). In total, 1433 distinct proteins were identified across the analyzed samples. Proteins associated with actin filaments or Z-discs accounted for 8 of the 10 most abundant proteins in nemaline rod areas of both myopathies (Additional file 2: Table 2).

Comparison of nemaline rod areas to rod-free areas across all specimens identified 142 differentially expressed proteins (Additional file 2: Table S3 and Fig. 2B). Proteins over-represented in rod areas included primarily nuclear proteins, proteins associated with the sarcolemmal membrane or extracellular matrix, and proteins associated with actin filaments or Z-discs. Protein sets related to extracellular matrix organization, DNA maintenance and replication, transcription and translation, and signaling cascades were differentially expressed. Comparisons of rod and rod-free areas performed separately in SLONM and in iNM each identified differential expression of nuclear, extracellular matrix, and actin filament proteins. Taken together, these findings point to alterations in basal and housekeeping mechanisms between the nemaline rod areas and rod-free areas.

Protein abundance in areas with nemaline rods was then compared between SLONM and iNM, identifying 51 differentially expressed proteins (Additional file 2: Table S4 and Fig. 2B). Mitochondrial proteins comprised the majority of proteins over-represented in SLONM, while sarcomeric proteins were the group most over-represented in iNM. Differentially expressed protein sets included those related immune, structural, metabolic and cellular pathways (Fig. 2C and Additional file 1: Fig. S1).

Analysis of protein abundance in the rod-free areas of SLONM and iNM biopsies identified differential expression of 10 proteins, including increased expression in SLONM of sarcomeric proteins associated with type II fibers (Additional file 2: Table S5 and Fig. 2B). There was also differential expression of proteins related to glycolysis and gluconeogenesis. No proteins showed altered expression both in nemaline rod and rod-free areas.

In all 5 patients with *ACTA1*-related iNM, we were able to detect signature peptides derived from the mutated protein. These included 4 missense mutations and one stop codon mutation resulting in an extended protein. In contrast, all 4 patients with *NEB*- or *TNNT1*-related iNM had splice site or truncating mutations, and no novel signature peptides were detected in these patients.

### Transcriptomic analysis identifies distinct gene expression signatures associated with iNM versus SLONM

**Fig. 3:**
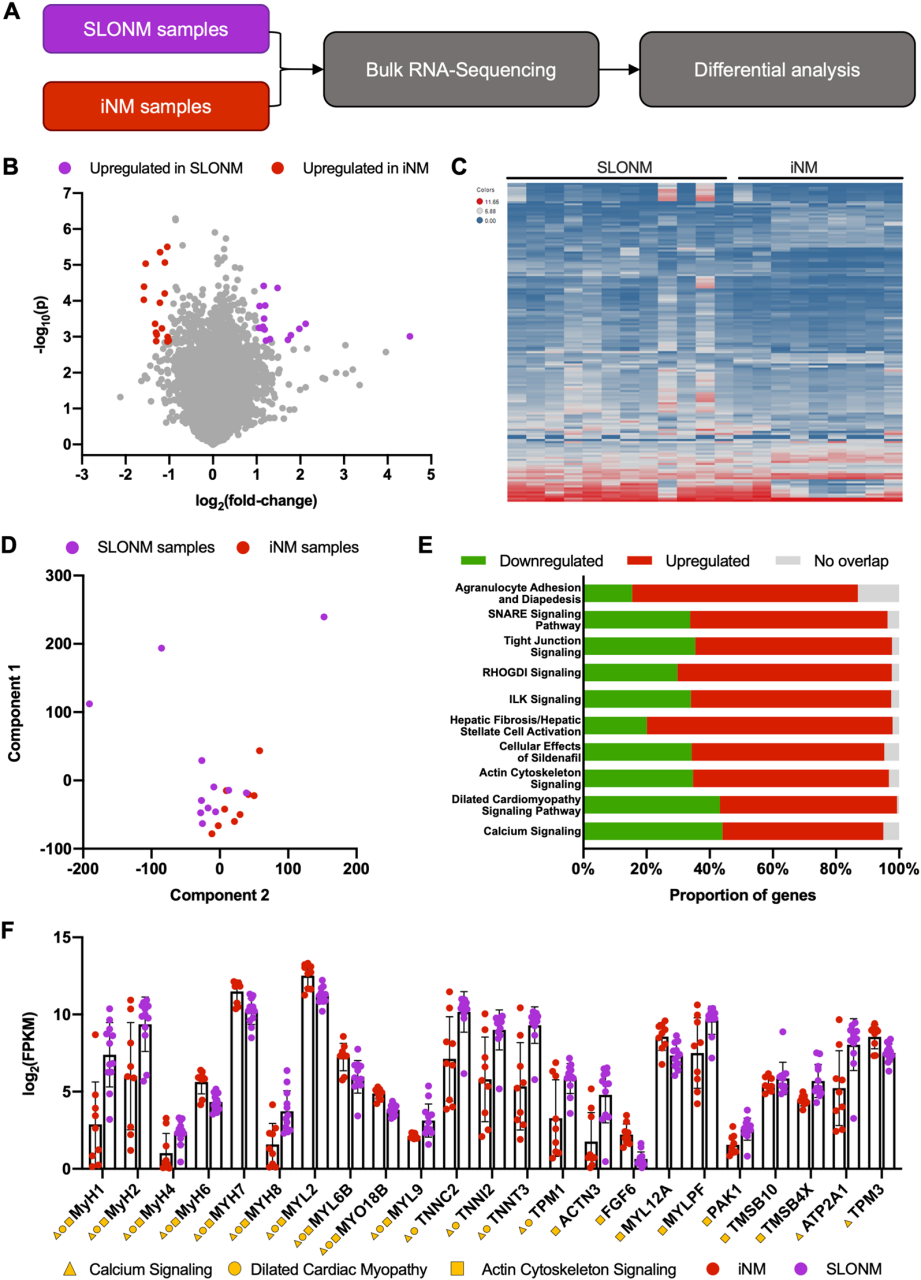
Transcriptomic analysis of inherited and sporadic late-onset nemaline myopathies (**A**) Bulk RNA sequencing was performed from sporadic late onset nemaline myopathy (SLONM) and inherited nemaline myopathy (iNM) muscle samples. (**B**) Transcriptomic analysis demonstrated 32 differentially expressed genes between the two disorders. (**C**) A heat map demonstrates the differential transcriptomic profiles of iNM and SLONM patient samples. (**D**) Principal component analysis showed differential clustering of iNM and SLONM transcriptomic profiles. (**E**) Ingenuity Pathway Analysis identified several pathways differentially expressed between the two disorders, the top 10 of which are illustrated here. (**F**) Differentially expressed genes in these pathways included several sarcomeric proteins, some of which were represented in multiple pathways. Bars represent mean ± SEM

Next, we performed bulk RNA sequencing on 24 samples (12 SLONM, 9 iNM, and 3 controls, Fig. 3A). This revealed 32 differentially expressed genes between the SLONM and iNM groups (Fig. 3B and C). Principal Component Analysis demonstrated differential clustering of the transcriptomic profiles of SLONM and iNM (Fig. 3D). Ingenuity Pathway Analysis identified pathways which were significantly altered between the two groups (Fig. 3E). The top 3 altered pathways were calcium signaling, dilated cardiomyopathy and actin cytoskeleton signaling. These were of greatest interest, as they are implicated in muscle physiology and disease. Several genes encoding sarcomeric proteins were represented in more than one of these pathways (Fig. 3F).

Transcriptomic analysis also identified 50 differentially expressed genes between SLONM and control samples (Additional file 1: Fig. S3A). These alterations included an increased expression of genes belonging to the antigen presentation pathway, the integrin-linked kinase (ILK) signaling pathway, calcium signaling and interferon signaling as the top 4 altered pathways (Additional file 1: Fig. S3B-D). Similarly, significant transcriptomic differences were observed between iNM and control samples (Additional file 1: Fig. S4A). The top 4 altered pathways were calcium signaling, dilated cardiomyopathy signaling, glycolysis and actin cytoskeleton signaling (Additional file 1: Fig. S4B–D).

### Integration of proteomics and transcriptomics reveals disease commonalities

Lastly, we compared the proteins differentially expressed in SLONM relative to iNM nemaline rod areas to genes showing significant differential expression in whole muscle transcriptomics. We found 3 targets which were common between the two datasets (*DPYSL3*, *MYH7*, *NRAP*), all of which were downregulated in SLONM relative to iNM (Additional file 1: Fig. S4).

### Multiple modalities show evidence of immunoglobulin deposition and immune activity

**Fig. 4:**
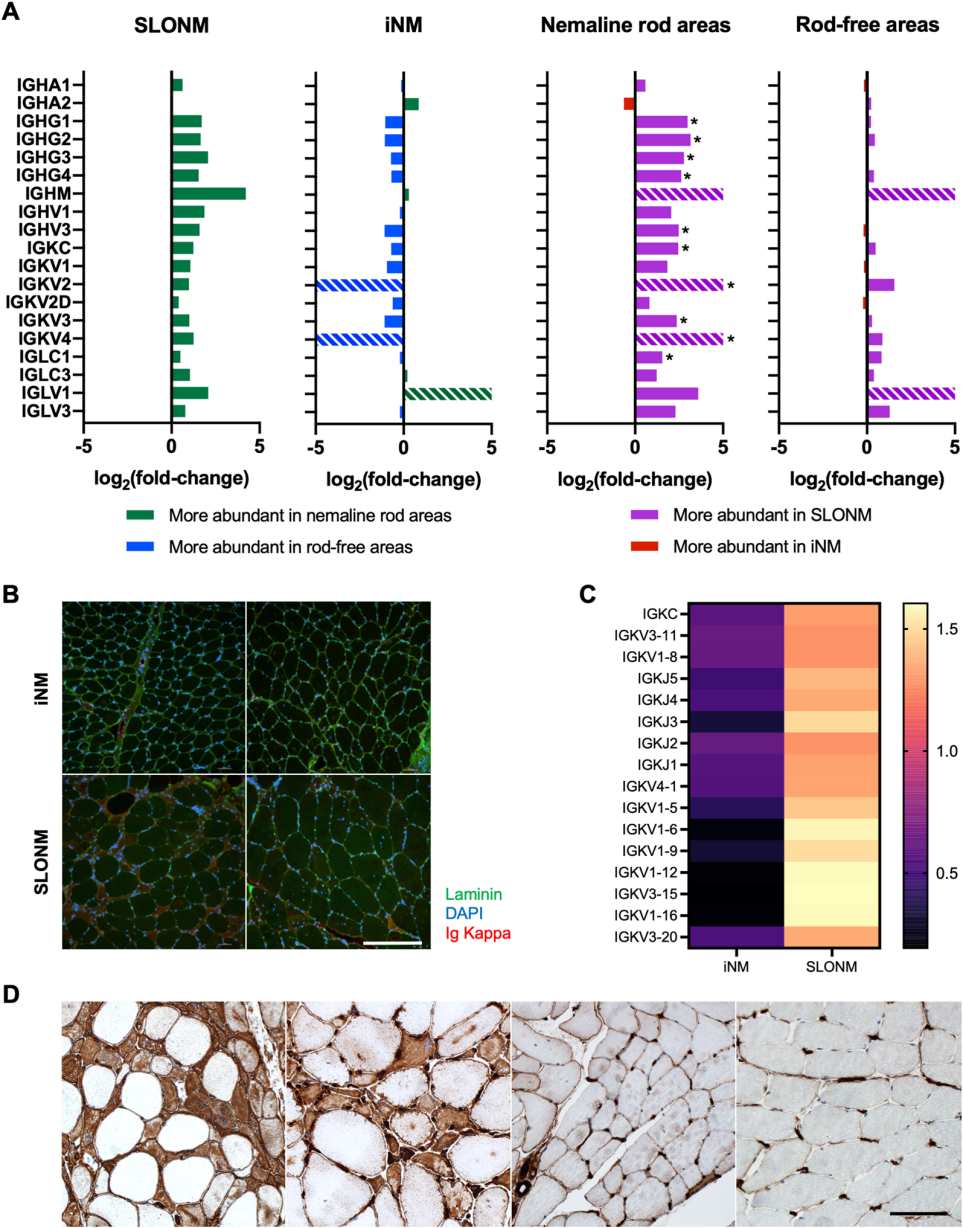
Analysis of immunoglobulin expression and autoimmune markers in inherited and sporadic late-onset nemaline myopathies (**A**) Proteomic analysis showed an increased abundance of IgG heavy chains and both kappa and lambda light chains in nemaline rod areas in sporadic late-onset nemaline myopathy (SLONM) relative to inherited nemaline myopathies (iNM). There was also a trend towards an increased abundance of immunoglobulin chains in the nemaline rod areas of SLONM relative to rod-free areas. Striped bars indicate immunoglobulin chains that were only detected in one of the groups of samples compared *p < 0.05. (**B**) Immunofluorescent staining demonstrated accumulation of kappa light chains in atrophic fibers in 38% of SLONM biopsies (bottom row), but not in iNM (top row). Scale bar = 400 µm. (**C**) Immunoglobulin genes were also differentially expressed between SLONM and iNM. (**D**) Immunostaining for Major Histocompatibility Antigen-I (MHC-I) showed reactivity in SLONM biopsies, occurring predominantly in atrophic fibers (first panel, SLONM with monoclonal gammopathy; second panel, SLONM without monoclonal gammopathy). Biopsies from patients with inherited nemaline myopathy showed no MHC-I reactivity or occasional fibers faintly reactive for MHC-I (third panel, adult patient with *ACTA1* nemaline myopathy). MHC-I reactivity was absent in healthy control muscle (fourth panel). Scale bar = 100 µm

Proteomic analysis of immunoglobulins revealed a significantly increased abundance of IgG HC, as well as kappa and lambda LC, in the nemaline rod areas of SLONM relative to iNM (Fig. 4A). There was also a trend towards an increased abundance of immunoglobulin chains in the nemaline rod areas of SLONM relative to rod-free areas. This difference met the significance threshold for Poisson FDR for 13 families of immunoglobulin chains but did not meet the more stringent quasi-likelihood FDR threshold, which takes into account observed variance in measurements. By contrast, there were no differences in immunoglobulin abundance between nemaline rod areas and rod-free areas in iNM, and no differences between rod-free areas in SLONM and iNM. Since immunoglobulins, especially variable regions, are naturally variable between patients, we further investigated and validated these findings through immunostaining. Diffuse kappa LC sarcoplasmic staining was observed in a proportion of atrophic fibers in 5 of 13 SLONM biopsies (1–17 fibers per high power field) and not observed in any of the 9 iNM biopsies (Fig. 4B). Among the 5 SLONM biopsies with kappa LC sarcoplasmic staining, 2 had an IgG kappa gammopathy, 2 had an IgG kappa gammopathy associated with a small IgG lambda gammopathy, and 1 had no monoclonal gammopathy at the time of biopsy. The latter patient however had a transiently elevated free light chain level prior to evaluation. The mRNA expression levels of immunoglobulin family members were also elevated in SLONM (Fig. 4C). We also immunostained the specimens for MHC-I, although MHC-I overreactivity can occur in the setting of immune-mediated but also inherited myopathies [31]. Among the 9 iNM specimens, 5 showed no MHC-I reactivity and 4 (all adults, age 31 to 73) showed occasional faintly reactive atrophic fibers. All SLONM specimens demonstrated muscle fibers with diffuse sarcoplasmic reactivity but in a variable number, predominantly in atrophic fibers but, in some samples, also in non-atrophic fibers (Fig. 4D). The sample with most kappa LC positive fibers had also the most MHC-I positive fibers (Fig. 4D, first panel).

## Discussion

SLONM and iNM share a key histological feature—nemaline rods—but they have vastly different etiologies and therapeutic implications. The distinction between these disorders generally rests on age of onset, disease course, genetic testing results, and presence of an underlying monoclonal gammopathy or HIV infection. We do note however that negative genetic testing cannot definitively exclude iNM, as a proportion of patients have non-coding and other mutations that are not readily detected by currently available clinical testing [32, 33]. In addition, approximately half of SLONM patients lack both monoclonal gammopathy and HIV infection. In these patients, there is no biomarker to confirm the diagnosis. In other patients, a monoclonal gammopathy may represent a coincidental finding, as monoclonal gammopathies of undetermined significance occur in 3% of individuals above age 50 [34]. This potential diagnostic uncertainty highlights the need to identify additional biomarkers of SLONM.

Pathological features of iNM have been extensively characterized [12, 35] and the concentration of rods in atrophic fibers in SLONM has been highlighted [9, 10, 36, 37], but a systematic pathological comparison between these two myopathies was lacking. Through quantitative and qualitative analysis of muscle biopsy samples, we identified a series of features that distinguish these two disorders. The histological hallmarks of SLONM included: nemaline rods in < 50% of muscle fibers, a smaller size of rod-containing fibers compared to fibers without rods, highly atrophic fibers filled with rods, necrotic fibers, regenerating fibers, and increased endomysial connective tissue. Conversely, most iNM biopsies showed nemaline rods frequently clustered subsarcolemmally or centrally, no or rare necrotic fibers, and unremarkable endomysial connective tissue, in keeping with previous reports [12]. In summary, the histological findings were distinct between the two myopathies. Our iNM cohort however did not include *TPM3*-related iNM cases where, due to restricted expression of alpha-tropomyosin-3 in type 1 fibers, rods are restricted to type 1 fibers [38, 39]. These can be hypotrophic and therefore can appear filled with rods, but the findings remain different than in SLONM, where atrophic fibers filled with rods are of either type. We also note that our iNM cohort did not include muscle biopsies from severe early onset disease, which can seldom show fibrotic and fatty changes [40]. Such cases however are easily distinguishable from SLONM and pose no diagnostic dilemma. A minimal inflammatory reaction, in keeping with previous reports [10, 37], was observed only in a single SLONM biopsy.

Proteomic analysis was performed in the nemaline rod areas of SLONM and iNM samples to highlight both shared and distinct constituents of nemaline rods in these disorders. Nemaline rods are known to be composed of Z-disc and thin filament proteins [41]. Our analysis did detect many of these proteins in high abundance in the nemaline rod regions. For example, actin, α-actinin, and tropomyosin are well-characterized nemaline rod proteins and they were the first, third, and fourth most abundant proteins, respectively, in the nemaline rod areas. These proteins however were not differentially expressed between SLONM and iNM, indicating that they are present in similar amounts in the nemaline rods of the two disorders. By contrast, myotilin (MYOT), synaptopodin-2 (SYNPO2), LIM domain binding 3 (LBD3), PDZ And LIM Domain 7 (PDLIM7), telethonin (TCAP), supervillin (SVIL), and nebulin-anchoring protein (NARP) are proteins implicated in actin filament assembly and Z-disc structure that were differentially expressed, suggesting that these proteins may underlie differences in the composition and formation of nemaline rods. In addition to these, extracellular matrix and nuclear proteins were over-represented in nemaline rod areas in our samples. A similar finding was observed in a recent proteomic analysis comparing rod-containing fibers from 5 SLONM patients to healthy control tissue [42]. Presence of extracellular matrix proteins accompanying the accumulation of Z-disc-related proteins was also shown by proteomic studies within protein aggregates in myotilinopathy and filaminopathy, two myofibrillar myopathies [43, 44].

When comparing nemaline rod areas of SLONM and iNM, we found increased expression of multiple mitochondrial proteins in SLONM. This may represent either a difference in mitochondrial content and distribution, or ectopic localization of mitochondrial proteins. These findings are supported by previous investigations showing that nemaline rod formation can occur not only as result of structural cell derangements, but also as result of metabolic stressors, including ATP depletion and heat shock [41, 45, 46]. Both proteomic analysis from rod-free areas and bulk RNA-seq additionally identified differential expression of a number of sarcomeric components (e.g. myosins and troponins) and enzymes involved in energy metabolism. These are in keeping with an increased proportion of type I muscle fibers in iNM, as has previously been reported [12].

Correlation of proteomic and transcriptomic profiles identified 3 common targets over-expressed in iNM relative to SLONM: *MYH7*, *DPYSL3*, and *NRAP*. The increased expression of myosin heavy-chain 7 (MYH7) is consistent with an increased proportion of type 1 fibers in iNM. Dihydropyrimidinase-like 3 (DPYSL3) and nebulin-related anchoring protein (NRAP) are both associated with actin filaments [47, 48]. NRAP is involved in myoblast fusion and myofibril assembly, and its accumulation has been detected in mouse and zebrafish models of *KLHL41*-related nemaline myopathy [47, 49, 50]. Downregulation of NRAP ameliorated the disease phenotype in zebrafish, suggesting that it could represent a therapeutic target in nemaline myopathy [49]. DPYSL3 is expressed in skeletal and cardiac muscles, but little is known about its function in these tissues [51]. A *DPYSL3* missense variant was however reported in association with amyotrophic lateral sclerosis and shown to impair axonal growth [52]. In contrast to the above, there were also many targets showing alterations in only protein or RNA expression. This imperfect correlation is expected, as the proteomic analysis focused on nemaline rod areas. As such, it would not be expected to detect changes in expression of proteins localized to other subcellular compartments, but it would identify changes in protein localization (i.e. concentration to nemaline rods) even in the absence of an overall change in gene expression.

Lastly, we identified through proteomic analysis an accumulation of immunoglobulins in the nemaline rod areas of SLONM. Immunostaining confirmed that kappa LC deposition is indeed present in a proportion of fibers in some SLONM samples (42%), but not in iNM, and may thus have value as a diagnostic marker. In keeping with this finding, transcriptomic analysis found increased expression of kappa LC genes in SLONM samples. To date, detection of immunoglobulins in SLONM muscle has been controversial and reported by some authors and not by others [9, 37, 53, 54]. The combination of our immunohistochemical, proteomic and transcriptomic findings supports the previously speculated pathogenic link between nemaline rods in SLONM and immunoglobulins, giving clinical significance to the monoclonal protein, which would no longer be an MGUS. Thus, it also provides further rationale for the use of plasma cell targeting chemotherapy for treatment of SLONM [55]. While all SLONM specimens showed MHC-I reactivity predominantly involving atrophic fibers, as previously reported [42], only 5 demonstrated diffuse kappa LC sarcoplasmic staining. Due to extreme heterogeneity of treatment (including intravenous immunoglobulins, methylprednisolone, methotrexate, mycophenolate mofetil, and autologous stem cell transplant) and variable length of follow-up, we were unable to assess whether patients with SLONM who had kappa LC-positive muscle fibers responded better to treatment. Comparison between a larger number of SLONM samples with and without monoclonal gammopathy would be needed to further clarify the role of the gammopathy.

One limitation of this study is the small number of samples available for analysis, and our findings would benefit from confirmation in a larger cohort. Because of the limited number of patients and genetic heterogeneity of iNM, we were unable to confidently establish features distinguishing different genetic subgroups of iNM. We also note that the majority of iNM patients in this cohort were aged < 18, and it is thus possible that a change in molecular characteristics may occur over time in some of these individuals. An additional uncorrected variable in our data is introduced by the different site of muscle biopsy in different patients. Although the biopsy was performed in a clinically and pathologically affected muscle, different human muscles have physiologically distinct gene signatures mainly due to the different combination of slow- and fast-twitch muscle genes expression and related metabolism. In the future, single-cell RNA-sequencing to characterize a more specific gene expression profile could circumvent this limitation [56]. Lastly, laser capture microdissection is unable to isolate structures as small as individual nemaline rods and therefore we isolated subcellular regions where rods were concentrated. Differentially expressed proteins in this analysis therefore represent both proteins localized to the rods themselves as well as adjacent regions.

## Conclusions

In summary, our findings represent a systematic examination of the pathological features of iNM and SLONM. This analysis identified a combination of histological features that distinguish the two disorders, and which may be valuable to clinical practice. We also identified proteins and genes that are differentially expressed between the two disorders, highlighting key molecular differences between SLONM and iNM and paving the way towards a more comprehensive understanding of underlying mechanisms of action, and eventually, precision therapies to better treat these disorders.

## Supplementary Information

**Additional file 1.** **Figure 1** Pathway enrichment analysis in proteomic data (A-D) Representative enrichment plots of 12 immune, structural, metabolic and cellular pathways. **Figure 2** Transcriptomic analysis demonstrates differences between control and SLONM patient samples (A) Top 10 significantly altered pathways between SLONM and control patient muscles (B-D) Log2 FPKM values for genes that were altered in the top 4 pathways. **Figure 3** Transcriptomics demonstrates differences between control and SLONM patient samples (A) Top 10 significantly altered pathways between iNM and control patients. (B-D) Log2 FPKM values for genes that were altered in the top 4 pathways. **Figure 4** Integration of transcriptomics and proteomics data demonstrates common targets (A) Venn diagram of proteins altered in the nemaline rod areas and genes altered in the whole muscle of SLONM vs iNM patients (B) Graph depicting protein and transcript expression of all analyzed targets in SLONM vs iNM, highlighting those showing significant differential expression in both analyses (C)Log2 FPKM levels and (D) Protein proportions of the 3 common genes.

Additional file 2 **Table 1** Genes tested. **Table 2** Proteins detected in nemaline rod areas. **Table 3** Proteins with different abundance in nemaline rod vs rod-free areas. **Table 4** Proteins with different abundance in nemaline rod areas in SLONM vs iNM. **Table 5** Proteins with different abundance in rod-free areas in SLONM vs iNM

## Data Availability

Proteomic data supporting the findings of this study have been deposited to MassIVE (https://massive.ucsd.edu/), a member of the ProteomeXchange consortium,  under the identifier MSV000090344. Transcriptomic data have been deposited in the Sequence Read Archive (https://www.ncbi.nlm.nih.gov/sra) under accession number PRJNA882425. Microscopy data reported in this paper are available from the corresponding author on reasonable request.

## Abbreviations

AGC: Automatic gain control
FDR: False discovery rate
ILK: Integrin-linked kinase
iNM: Inherited nemaline myopathy
HC: Heavy chain
LC: Light chain
maxIT: Maximum ionization time
MGUS: Monoclonal gammopathy of unknown significance
nLC-MS/MS: Nano-scale liquid chromatography tandem mass spectrometry
SLONM: Sporadic late onset nemaline myopathy
Th: Thompson
