# Supplementary material for: Molecular signatures of inherited and acquired sporadic late onset nemaline myopathies
Source: Acta Neuropathol Commun. 2023 Jan 26;11:20. doi: 10.1186/s40478-023-01518-9 (PMC9878979; doi:10.1186/s40478-023-01518-9)
Supplement: Supplementary file 1 — Additional file 1. Figure 1 Pathway enrichment analysis in proteomic data (A-D) Representative enrichment plots of 12 immune, structural, metabolic and cellular pathways. Figure 2 Transcriptomic analysis demonstrates differences between control and SLONM patient samples (A) Top 10 significantly altered pathways between SLONM and control patient muscles (B-D) Log2 FPKM values for genes that were altered in the top 4 pathways. Figure 3 Transcriptomics demonstrates differences between control and SLONM patient samples (A) Top 10 significantly altered pathways between iNM and control patients. (B-D) Log2 FPKM values for genes that were altered in the top 4 pathways. Figure 4 Integration of transcriptomics and proteomics data demonstrates common targets (A) Venn diagram of proteins altered in the nemaline rod areas and genes altered in the whole muscle of SLONM vs iNM patients (B) Graph depicting protein and transcript expression of all analyzed targets in SLONM vs iNM, highlighting those showing significant differential expression in both analyses (C)Log2 FPKM levels and (D) Protein proportions of the 3 common genes. [file 40478_2023_1518_MOESM1_ESM.pdf]

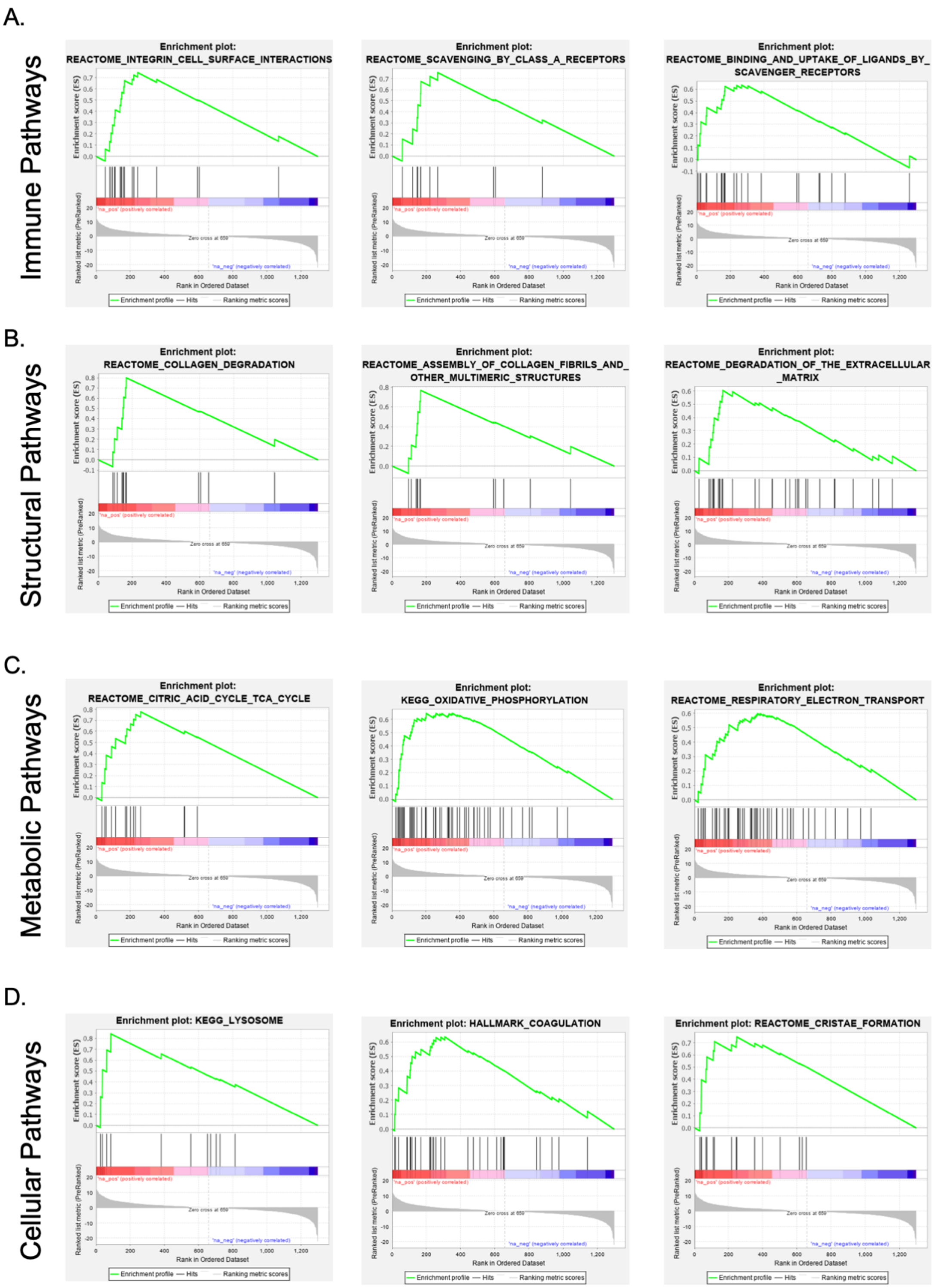

Supplementary Figure 1: Pathway enrichment analysis in proteomic data

(A-D) Representative enrichment plots of 12 immune, structural, metabolic and cellular pathways

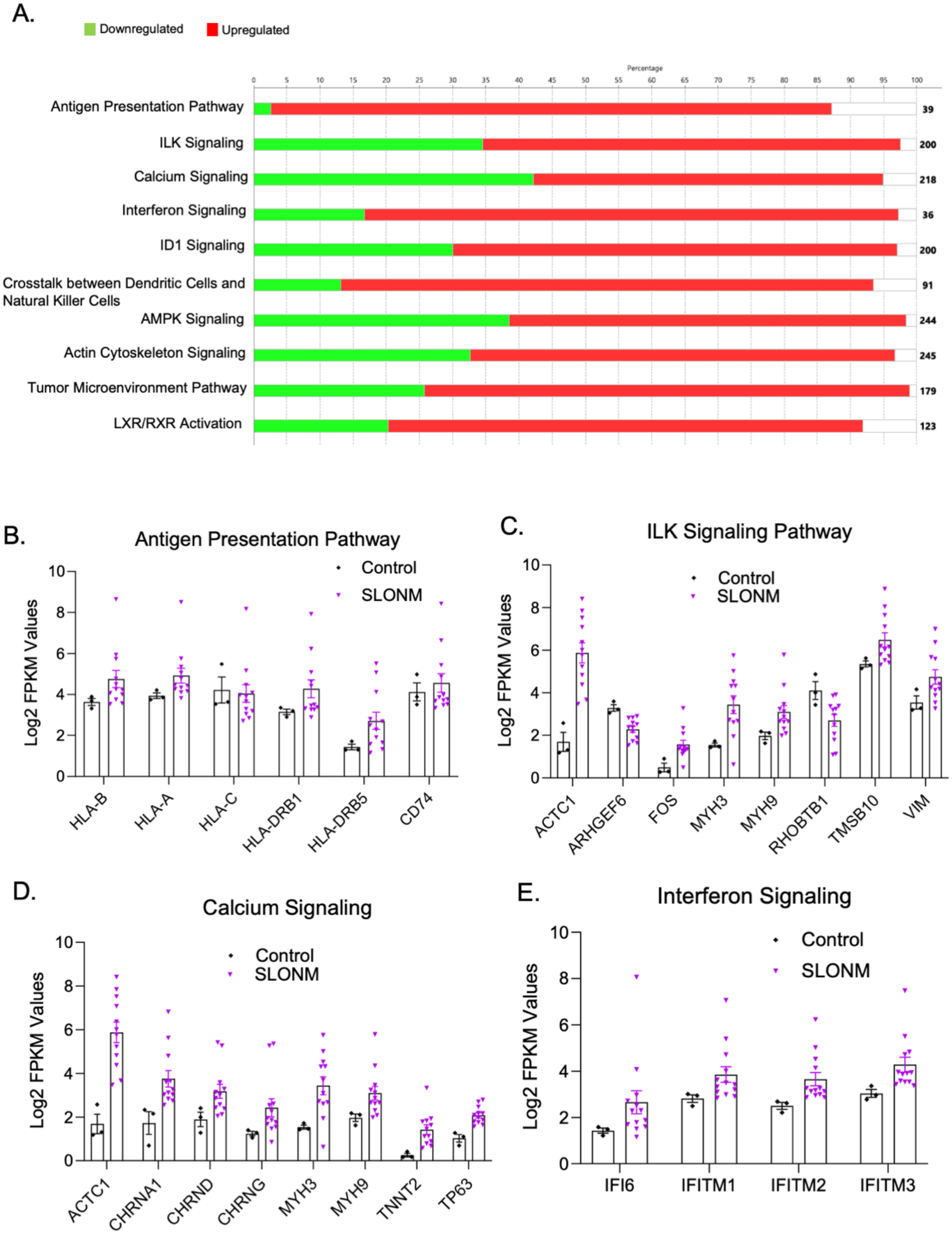

Supplementary Figure 2: Transcriptomic analysis demonstrates differences between control and SLONM patient samples

(A) Top 10 significantly altered pathways between SLONM and control patient muscles
(B-D) Log2 FPKM values for genes that were altered in the top 4 pathways.

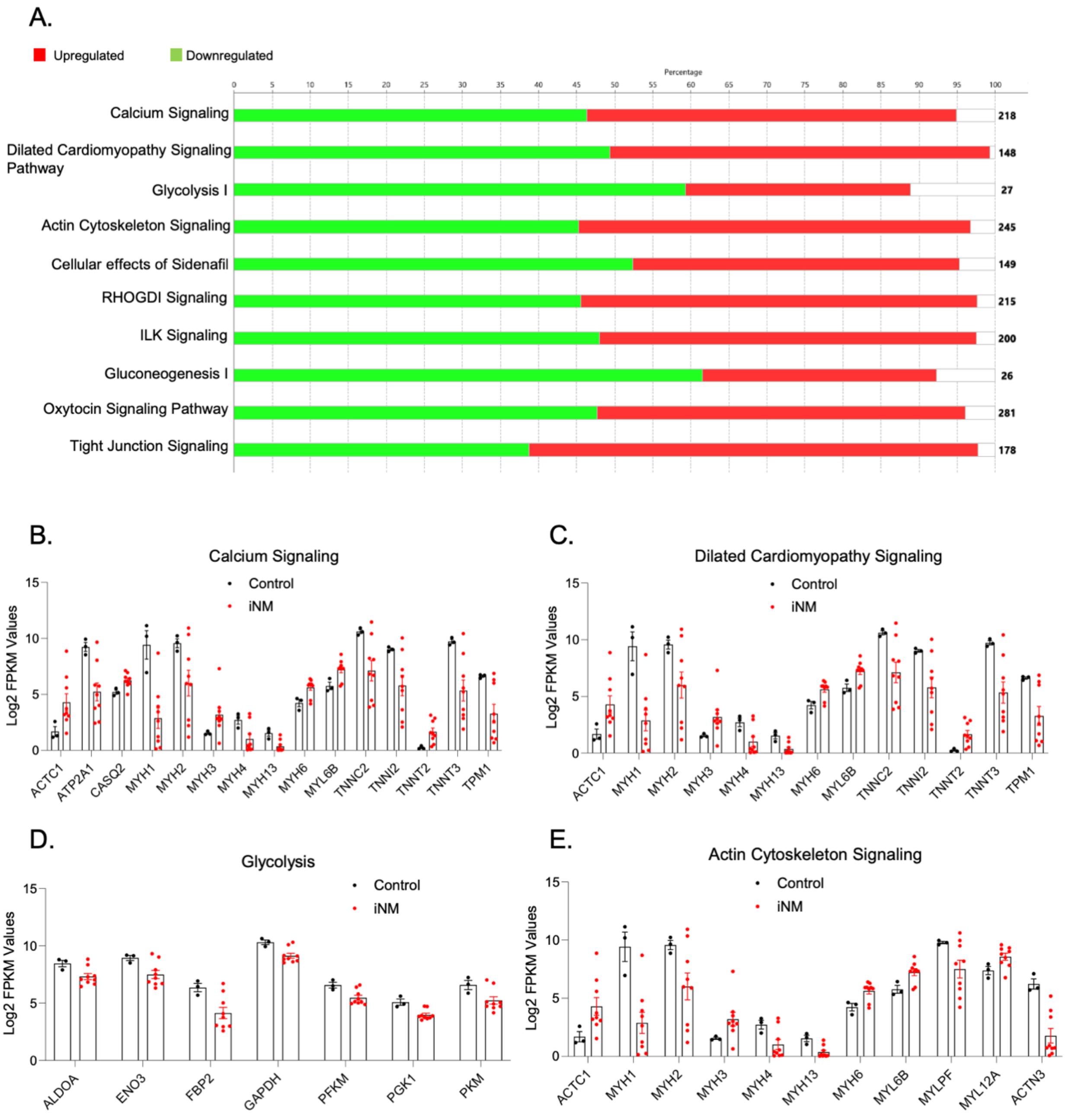

Supplementary Figure 3: Transcriptomics demonstrates differences between control and SLONM patient samples

(A) Top 10 significantly altered pathways between iNM and control patients. (B-D) Log2 FPKM values for genes that were altered in the top 4 pathways.

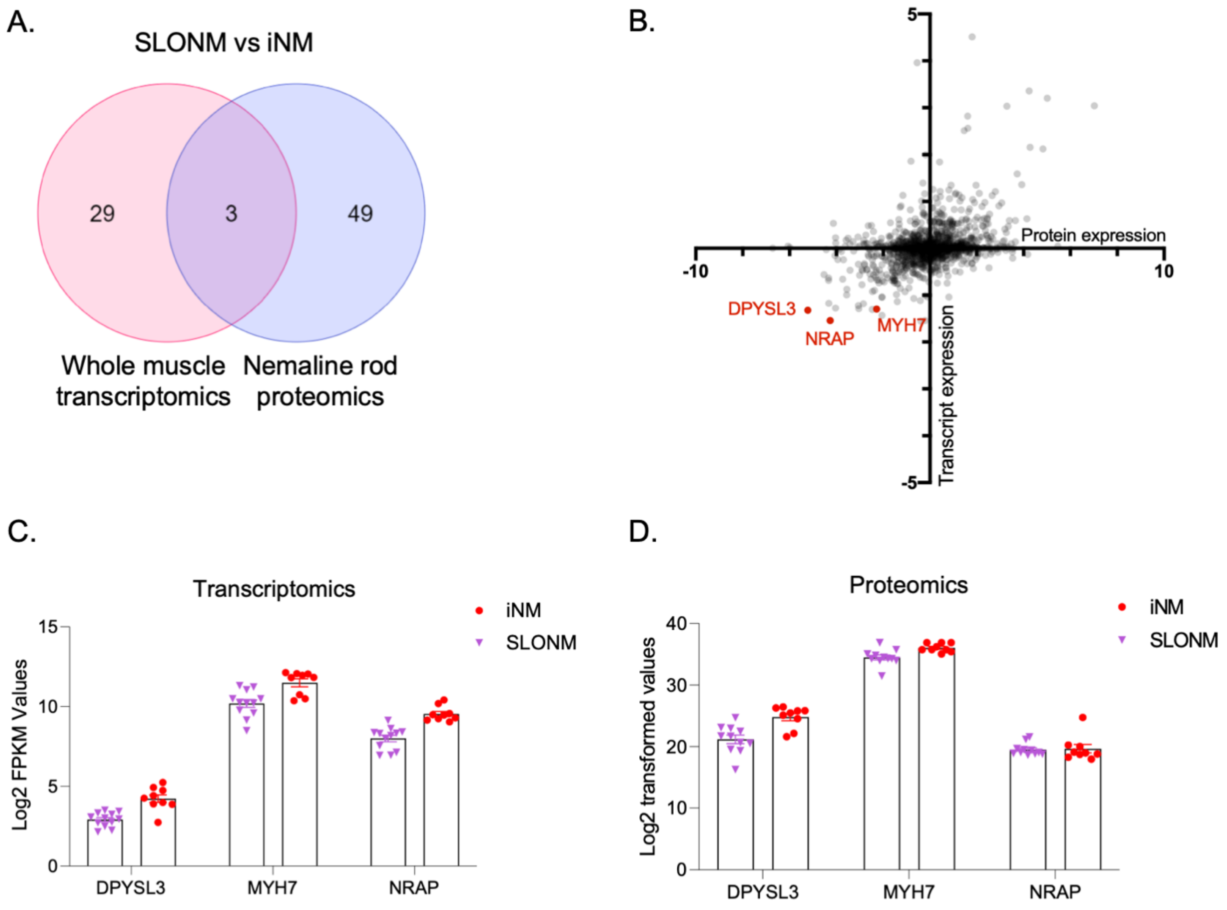

Supplementary Figure 4: Integration of transcriptomics and proteomics data demonstrates common targets

(A) Venn diagram of proteins altered in the nemaline rod areas and genes altered in the whole muscle of SLONM vs iNM patients (B) Graph depicting protein and transcript expression of all analyzed targets in SLONM vs iNM, highlighting those showing significant differential expression in both analyses (C)Log2 FPKM levels and (D) Protein proportions of the 3 common genes.
